# Supplementary material for: Development of a computer-aided design software for the quantitative evaluation of aesthetic damage
Source: PLoS One. 2019 Dec 18;14(12):e0226322. doi: 10.1371/journal.pone.0226322 (PMC6919621; doi:10.1371/journal.pone.0226322)
Supplement: S1 Code site — 1. (ZIP) [file pone.0226322.s001.zip › [EESC_jr][Projeto]Relatorio_07_21/reports/templates_zip/gentelella-master/vendors/jszip/documentation/examples/download-zip-file.html]

---
title: "Download the generated zip file"
layout: default
section: example
---

Tip : check the source of the page !

## The FileSaver API

Works on firefox, chrome , opera >= 15 and IE >= 10 (but NOT in compatibility view).  
click to download

## The data URL

Does not work in IE, has restrictions on the length.  
click to download
